# Conserving Biodiversity in a Human-Dominated World: Degradation of Marine Sessile Communities within a Protected Area with Conflicting Human Uses

**DOI:** 10.1371/journal.pone.0075767

**Published:** 2013-10-15

**Authors:** Valeriano Parravicini, Fiorenza Micheli, Monica Montefalcone, Carla Morri, Elisa Villa, Michela Castellano, Paolo Povero, Carlo Nike Bianchi

**Affiliations:** 1 CESAB-FRB, Immeuble Henri Poincaré - Domaine du Petit Arbois, Aix-en-Provence, France; 2 IRD – UR 227 Coreus – Laboratoire Arago, Banyuls/mer, France; 3 Hopkins Marine Station, Stanford University, Pacific Grove, California, United States of America; 4 DiSTAV, Dipartimento di Scienze della Terra, dell'Ambiente e della Vita, Genoa University, Genoa, Italy; CSIR- National institute of oceanography, India

## Abstract

Conservation research aims at understanding whether present protection schemes are adequate for the maintenance of ecosystems structure and function across time. We evaluated long-term variation in rocky reef communities by comparing sites surveyed in 1993 and again in 2008. This research took place in Tigullio Gulf, an emblematic case study where various conservation measures, including a marine protected area, have been implemented to manage multiple human uses. Contrary to our prediction that protection should have favored ecosystem stability, we found that communities subjected to conservation measures (especially within the marine protected area) exhibited the greatest variation toward architectural complexity loss. Between 1993 and 2008, chronic anthropogenic pressures (especially organic load) that had already altered unprotected sites in 1993 expanded their influence into protected areas. This expansion of human pressure likely explains our observed changes in the benthic communities. Our results suggest that adaptive ecosystem-based management (EBM), that is management taking into account human interactions, informed by continuous monitoring, is needed in order to attempt reversing the current trend towards less architecturally complex communities. Protected areas are not sufficient to stop ecosystem alteration by pressures coming from outside. Monitoring, and consequent management actions, should therefore extend to cover the relevant scales of those pressures.

## Introduction

Most marine ecosystems are challenged worldwide by a vast set of human pressures ranging from pollution to habitat modification, overfishing and climate change [1]. The recognition of the profound influence humans exert on marine ecosystems has motivated worldwide conservation efforts mainly aimed at the establishment of Marine Protected Areas (MPAs). Yet, MPAs alone are not sufficient [2], [3]. They rarely cover an adequate extent of ecosystem types being typically quite small (ranging approx. 10^−3^ to 10^3^ km^2^) and are thereby ineffective in halting the effects of pressures acting at scales larger than those encompassed by the protection schemes.

Anthropogenic pressures acting over large scales are common [1], especially along intensely populated coastal areas, where marine ecosystems are challenged by the cumulative effect of multiple pressures and conflicting human uses. The need for complementary measures to the protection by MPAs has called for the development of ecosystem-based management (EBM) to be implemented at regional scales. EBM is an integrated approach that considers the entire ecosystem, including humans [4]. EBM differs from other approaches that focus on a single species or sector because it includes consideration of the interactions among ecosystem components and the cumulative impacts of multiple activities [5]. Some examples of EBM implementation are the zoning of the Great Barrier Reef Marine Park [6] and the establishment of marine sanctuaries in the USA [7]. Similar instruments have been developed in the European Union, where legislation mandates the establishment of a network of *Sites of Community Importance* (SCIs) to preserve habitats and species [8] and *Integrated Coastal Zone Management* (ICZM), whose goal is the sustainable development of coastal areas [9]. Despite the application of EBM approaches to complement MPAs, few studies have assessed whether such plans effectively sustain the conservation of marine coastal ecosystems. Ecosystem-based approaches have recently been advocated also to understand ecosystem shifts due to climate change [10].

Thanks to historical data from 1993 onwards ([11] and unpublished information), we were able to evaluate the results of the management plan applied in an emblematic study case (the Tigullio Gulf) by examining the decadal-scale change of rocky reef communities. The Tigullio Gulf is a densely populated area where conservation measures included the establishment of one MPA in 1998 (designed mainly to regulate fishing) and of six SCIs in 2000 (designed to regulate multiple human pressures on protected habitat and species). The area has been, and still is, subjected to various human uses and associated possible impacts: organic load from sewage outfalls, input of sediments and nutrients from a river, aquaculture, periodic beach replenishments, and small marinas.

Coastal development and related pressures have been shown to affect Mediterranean rocky reef communities by causing the replacement of canopy-forming algae with algal turfs, with consequent habitat homogenization and loss of architectural complexity [12]. In addition to removing or preventing these disturbances directly, through a regulation of development within SCIs, MPAs are expected to maintain or recover algal canopies through top-down control as protection from fishing allows predatory fishes to exert control upon sea urchins, which otherwise may proliferate and overgraze benthic algae [13], [14]. Yet, at the same time, protection in MPAs may increase the abundance of herbivorous fish, which may also imply increased grazing pressure on canopy-forming algae [15].

To date, few studies have addressed how concomitant human pressures [16] impact reef communities within the Mediterranean Sea and whether EMB effectively manages non-fishing human uses that are not reduced by MPAs.

Here, we compared surveys of reef communities conducted prior to protection in 1993 and in 2008, 10 years after MPA and 8 years after SCIs establishment. We assessed relevant pressures operating in the study area and we related their intensities and trends to the observed change in reef communities in order to clarify whether the existing protection effort was effective. Results represent the first assessment of the efficacy of current management plans, and provide guidelines for EBM, with particular attention to the design of monitoring plans.

## Methods

### Study area and pressure regime

**Figure 1:**
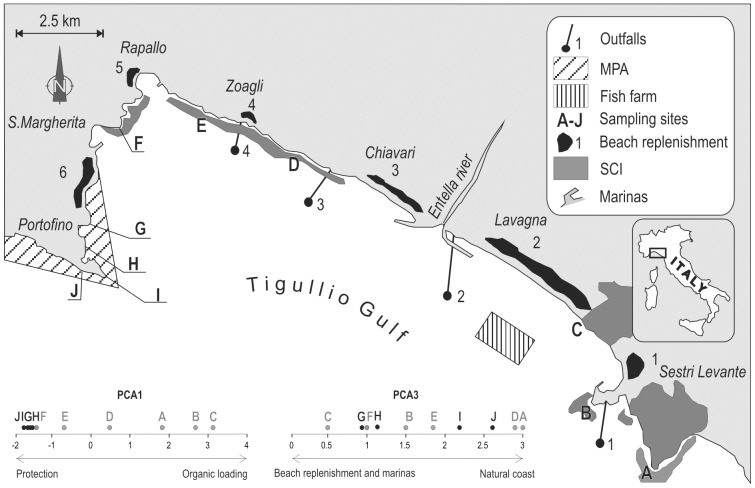
Position of sampling sites and of the main human-induced pressures in the study area. On the bottom, the position of individual sites on PCA axes is shown. The first axis represents closeness to sources of organic loading, the third closeness to replenished beaches and marinas.

The Tigullio Gulf is located in the Ligurian Sea, NW Mediterranean Sea (Figure 1). The coastal zone is partially included within the Portofino MPA and six SCIs [3]. According to the standards of the European Water Framework Directive, water quality in the study area is classified as ‘good’ [17]. However, the area is densely populated by residents and tourists, resulting in comparatively high nutrient inputs into coastal waters.

Since the late 1950s, many studies have been carried out in this area [18]. Historical information on planktonic production [19], [20] evidenced that chlorophyll-*a* concentration was significantly lower in the period 2000–2008 than in 1985–1994 (Figure S1a; t-test, df = 8, *P*<0.017). On the contrary, water transparency did not show any significant variation of the annual mean for the same periods (Figure S1b).

Potential anthropogenic pressures on coastal marine ecosystems in the last two decades include the presence of four outfalls discharging above the summer thermocline (i.e. about 25 m depth), the Entella River mouth (with a number of waste water pipes along its way), a fish farm (established in 2000), marinas and tourist harbors (built during the 1970s), and beaches frequently replenished (Table S1). Human population remained virtually stable: residents passed from 52 inhabitants km^−2^ in 1993 to 50 inhabitants km^−2^ in 2008, tourists from 1.34 million in 1998 to 1.36 million in 2007 (ISTAT, the Italian Statistical Institute). Due to change in local industrial activity, the potential organic load computed in term of equivalent individuals [21] showed a slight increase from 1991 to 2001 (Figure S1c). In the same decades, global warming has been showing a large influence on the hydrology and ecology of the Mediterranean basin [22]; the Ligurian Sea, in particular, underwent a regime shift in the late 1990s, two major heat waves in 1999 and 2003 having caused mass mortalities of gorgonians, sponges and other sessile organisms [23]–[25].

### Sampling

Reef communities were surveyed in summer 1993 [11], before the establishment of the MPA and SCIs, in 10 sites showing similar environmental characteristics (Figure 1). Four sites (G, H, I and J) have subsequently been included within the Portofino MPA in 1998, the remaining six sites within SCIs since 2000. When necessary, all permits needed for performing fieldwork were obtained by the Genoa University from the Portofino MPA Authority. At each site, the percent cover of individual epibenthic taxa was visually estimated within four 1 m^2^ quadrats randomly placed on vertical rocky walls at 5 m depth. Although the sampling effort employed may be considered rather small, Mediterranean rocky reef communities are ‘miniaturized’ compared to those of other temperate regions, and the surface of epibenthic communities surveyed at each site is one order of magnitude larger than the recommended minimum area of 2,170 cm^2^ to be sampled in the Mediterranean [26]. Thus, our sampling units encompassed small-scale heterogeneity, while the four replicates were placed within a habitat (shallow vertical and sub-vertical walls) that exhibits fairly homogeneous assemblages over scales of 10 s m.

Percent cover was quantified by dividing the sampling quadrat into 25 sub-squares through a nylon line and giving each taxon a score from 0 to 4 within each square and then adding up scores for all squares within the quadrat [27]. Species identification was conducted visually at the lower possible taxonomic level; voucher specimens were collected only when in doubt and never involved protected or endangered species. In the same quadrats used for the assessment of epibenthic communities, the total density of sea-urchins was estimated. In 2008, sites were precisely relocated by two of the authors (CNB and CM) that sampled communities in 1993 [11]. The sites were then surveyed by relocating 4 sampling quadrats at 5 m depth and following the same procedure. Vertical rocky walls were about 10 m height and the tidal height in the area is about 30 cm.

### Community descriptors

Architectural aspects were considered in addition to taxonomic composition. For architectural complexity [28], the three-dimensionality of the assemblages was assessed. Based on the ratio between height (h) and radius (r), species were grouped into five categories: ‘high’ (h≫r, e.g. *Cystoseira zosteroides*), ‘medium-high’ (h>r, e.g. *Dictyopteris polypodioides*), ‘medium’ (h∼r, e.g. *Reteporella grimaldii*), ‘medium-low’ (h<r, e.g. *Petrosia ficiformis*), and ‘low’ (h≪r, e.g. *Lithophyllum incrustans*).

### Data analysis

In order to characterize the degree of human pressure upon individual sites, the distances from the MPA (protection) and the main potential sources of impact (i.e. river mouth, outfalls, fish farm, marinas, and replenished beaches) were calculated for each site. As raw explanatory variables were collinear, the matrix of distances from potential impact sources was treated with principal component analysis (PCA) [29]: the 1^st^ and the 2^nd^ axes of the PCA (PCA1 and PCA2) were found to be a good synthesis of the pressure gradient from protection by the MPA and closeness to potential sources of organic load (i.e. outfalls, river mouth, and fish farm), whereas the 3^rd^ axis was correlated with the distance from the marinas and the replenished beaches (Table S2).

Two distinct datasets were obtained from communities surveys, the first using species as variables, the second using categories of three-dimensional structure. In order to test whether a significant change occurred in the time considered, permutational multivariate analysis of variance (PERMANOVA) [30] was used on each dataset considering sampling year and sampling site as random, crossed factors. PCA1 and PCA2 were included in the analysis as covariates and their interaction with sampling year were considered in order to test whether different pressures lead to different variation through time. The two sampling years (i.e. 1993 vs. 2008) were considered as random. Protection in the MPA was not included as a categorical factor to avoid redundancy since its potential effect was already considered in the PCA1 as distance from the MPA.

Permutational analysis of multivariate dispersion (PERMDISP) was used to test potential variation in β diversity [31]. Multivariate patterns were visualized through non-metric multidimensional scaling ordination (nMDS). Similarity percentage analysis (SIMPER) [32] was used to identify the taxa and community descriptors mainly responsible for significant differences.

Sea-urchins had low abundance in both sampling occasions, and potential differences through time were assessed using descriptive statistics.

## Results

A total of 88 conspicuous sessile species was found (Table 1). The most speciose groups were algae (35 species, of which 17 rhodophytes, 7 ochrophytes, and 11 chlorophytes) and sponges (22 species); other invertebrate groups were less represented (9 cnidarians, 4 mollusks, 5 serpuloideans, 2 barnacles, 6 bryozoans, and 5 ascidians). Sessile communities had changed significantly between 1993 and 2008 (Table 2). Total species richness passed from 68 species (17±0.78 SE per quadrat) in 1993, to 81 (21±0.63 SE per quadrat) in 2008. Dominant species in 1993 included tall algae such as *Dictyopteris polypodioides* and *Sphaerococcus coronopifolius*, which had nearly disappeared in 2008; amongst the invertebrates, sponges decreased, while hydroids increased.

**Table 1:** Total list of the sessile taxa found, in systematic order by major taxa.

**Ochrophyta**	*Crambe crambe*
*Cystoseira zosteroides*	*Dysidea avara*
*Dictyopteris polypodioides*	*Haliclona cratera*
*Dictyota dichotoma*	*Haliclona fulva*
*Dictyota fasciola*	*Hemimycale columella*
*Dictyota implexa*	*Hymeniacidon perlevis*
*Padina pavonica*	*Ircinia oros*
*Stypocaulon scoparium*	*Ircinia variabilis*
**Rhodophyta**	*Leucosolenia variabilis*
*Acrothamnion preissii*	*Petrosia ficiformis*
algal turf	*Phorbas tenacior*
*Amphiroa rigida*	*Spongia lamella*
*Amphiroa cryptarthrodia*	**Cnidaria**
*Asparagopsis armata*	*Aiptasia mutabilis*
*Bonnemaisonia asparagoides*	*Balanophyllia europaea*
*Ellisolandia elongata*	*Caryophyllia inornata*
*Jania rubens*	*Cladocora caespitosa*
*Laurencia obtusa*	*Clavularia crassa*
*Lithophyllum incrustans*	*Eudendrium racemosum*
*Lithophyllum stictaeforme*	Hydrozoa indet.
*Mesophyllum lichenoides*	*Parazoanthus axinellae*
*Peyssonnelia rubra*	*Pennaria disticha*
*Peyssonnelia squamaria*	**Mollusca**
*Sphaerococcus coronopifolius*	*Arca noae*
*Tricleocarpa fragilis*	*Chama gryphoides*
*Wrangelia penicillata*	*Thylacodes arenarius*
**Chlorophyta**	*Vermetus triquetrus*
*Acetabularia acetabulum*	**Serpuloidea**
*Caulerpa racemosa*	*Protula tubularia*
*Cladophora prolifera*	*Salmacina dysteri*
*Cladophora* sp.	*Serpula vermicularis*
*Codium bursa*	Serpulidae indet.
*Codium coralloides*	Spirorbidae indet.
*Codium fragile*	**Cirripedia**
*Flabellia petiolata*	*Balanus trigonus*
*Halimeda tuna*	*Perforatus perforatus*
*Pseudochlorodesmis furcellata*	**Bryozoa**
*Valonia utricularis*	*Margaretta cereoides*
**Porifera**	*Reptadeonella violacea*
*Acanthella acuta*	*Reteporella grimaldii*
*Agelas oroides*	*Schizoporella dunkeri*
*Axinella damicornis*	*Schizoporella errata*
*Axinella polypoides*	*Scrupocellaria reptans*
*Axinella verrucosa*	**Ascidiacea**
*Chondrilla nucula*	*Clavelina lepadiformis*
*Chondrosia reniformis*	*Didemnum candidum*
*Clathrina clathrus*	*Diplosoma listerianum*
*Clathrina contorta*	*Halocynthia papillosa*
*Cliona viridis*	*Microcosmus polymorphus*

Within major taxa, species or species groups are ordered alphabetically. Species are named according to the World Register of Marine Species (www.marinespecies.org/).

**Table 2:** Results of PERMANOVA analyses on individual taxa and three-dimensional categories.

Taxonomic composition					
Source of variation	df	SS	MS	pseudo-F	*P* (perm)
Year	1	20266	20266	16.59	0.001
Site	9	53914	6739	5.51	0.001
PCA1	1	21050	21050	17.23	0.001
PCA3	1	2116	2116	1.73	0.075
Year×Site	9	27053	3381	2.76	**0.001**
Year×PCA1	1	5642	5641	4.61	**0.001**
Year×PCA3	1	580	580	0.47	0.902
Residuals	60	70853	1221		
Total	79	201440			

Three-dimensional structure					
Source of variation	df	SS	MS	pseudo-F	*P* (perm)
Year	1	1118	1118	4.66	0.008
Site	9	20121	2515	10.48	0.001
PCA1	1	11193	11193	46.63	0.001
PCA3	1	526	526	2.19	0.090
Year×Site	9	3788	473	1.97	**0.017**
Year×PCA1	1	2313	2313	9.64	**0.001**
Year×PCA3	1	214	214	0.89	0.424
Residuals	60	13920	240		
Total	79	53197			

Bold numbers indicate significant results.

**Figure 2:**
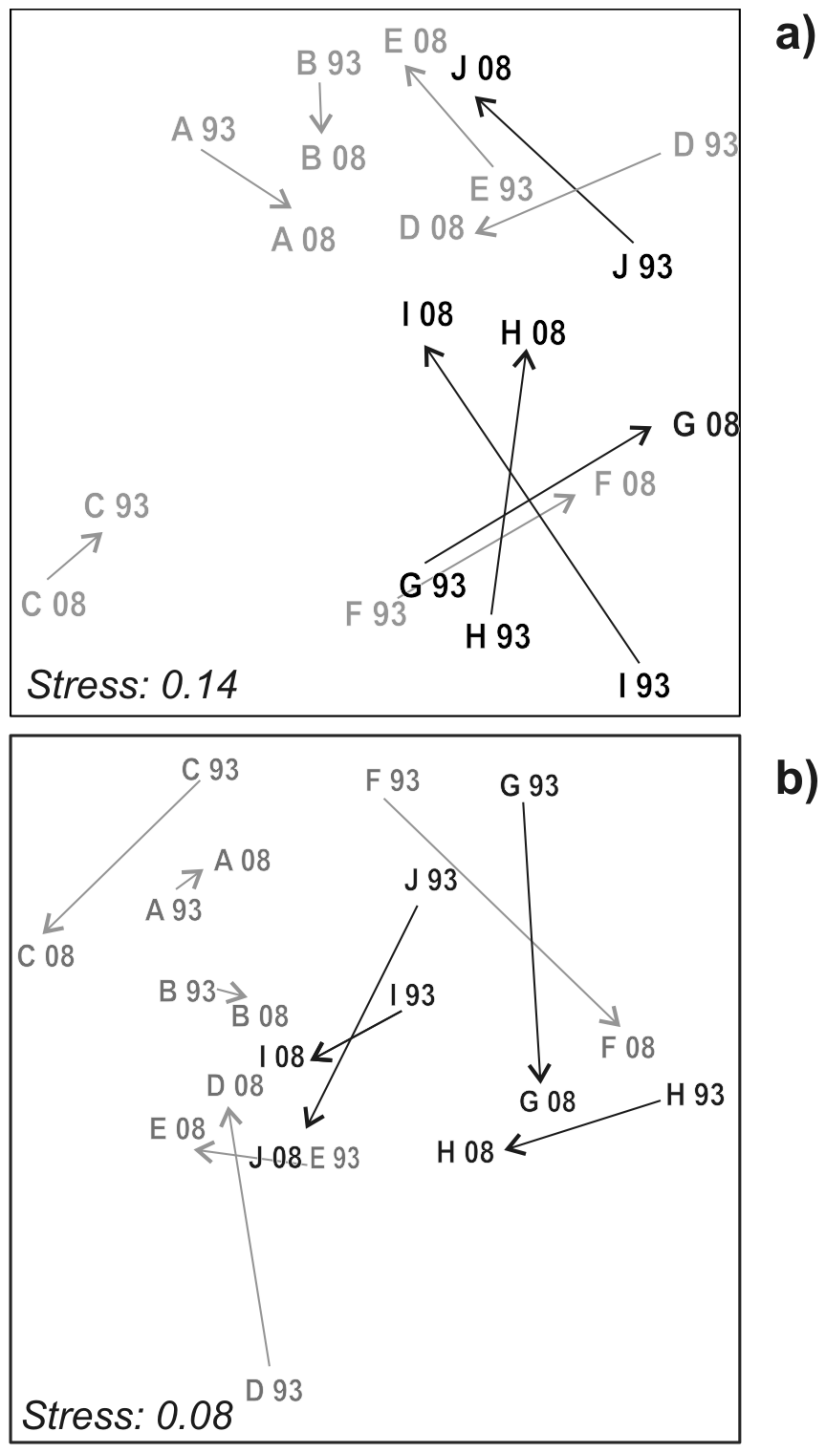
MDS plots calculated on species (a) and three-dimensional categories (b). Black characters and arrows refer to sampling sites inside the MPA, grey ones to sampling sites outside the MPA.

However, differences between the two sampling years were not consistent among sampling sites (significant Year×Sites) and were affected by the spatial distribution of human pressures, both for taxonomic composition and three-dimensional structure. In particular, the pressures considered by the PCA1 (i.e. protection by the MPA and distance from potential source of organic loading) had a significant role in driving the community change through time (significant Year×PCA1) whereas beach replenishments and tourist harbors played a negligible role (Year×PCA3 not significant). Correspondingly, the MDS plots highlighted marked differences in the direction of change between years (i.e. 1993 vs 2008) among individual sites using both species (Figure 2a) and categories of species three-dimensionality (Figure 2b) as community descriptors. MDS further suggested that sites far from potential sources of organic load or included within the MPA showed a higher degree of change between the two years than the remaining sites.

Although varying significantly among sites, the overall direction of change implied a decreased multivariate dispersion in the pattern of species occurrence and abundance among sites and hence a reduction in β diversity (PERMDISP: *P* = 0.04), indicative of homogenization of the communities through time.

Even within the MPA, the between-year change greatly varied among individual sites (SIMPER results, Table S3). *Dictyopteris polypodioides*, a potential canopy forming algal species, disappeared from site J (the furthest from potential impact sources) while *Dictyota dichotoma*, an erect generalist algal species, increased. The perennial algae *Halimeda tuna*, *Peyssonnelia squamaria* and *Flabellia petiolata* decreased at sites I, H and G, respectively, while the seasonal algae *Stypocaulon scoparium* and *Dictyota dichotoma* increased. Algal turf greatly increased at all sampling sites. The invasive algae *Caulerpa racemosa* and *Acrothamnion preissii* were recorded in 2008, but not in 1993. In particular, *Caulerpa racemosa* appeared only at sites close to potential sources of organic load.

**Figure 3:**
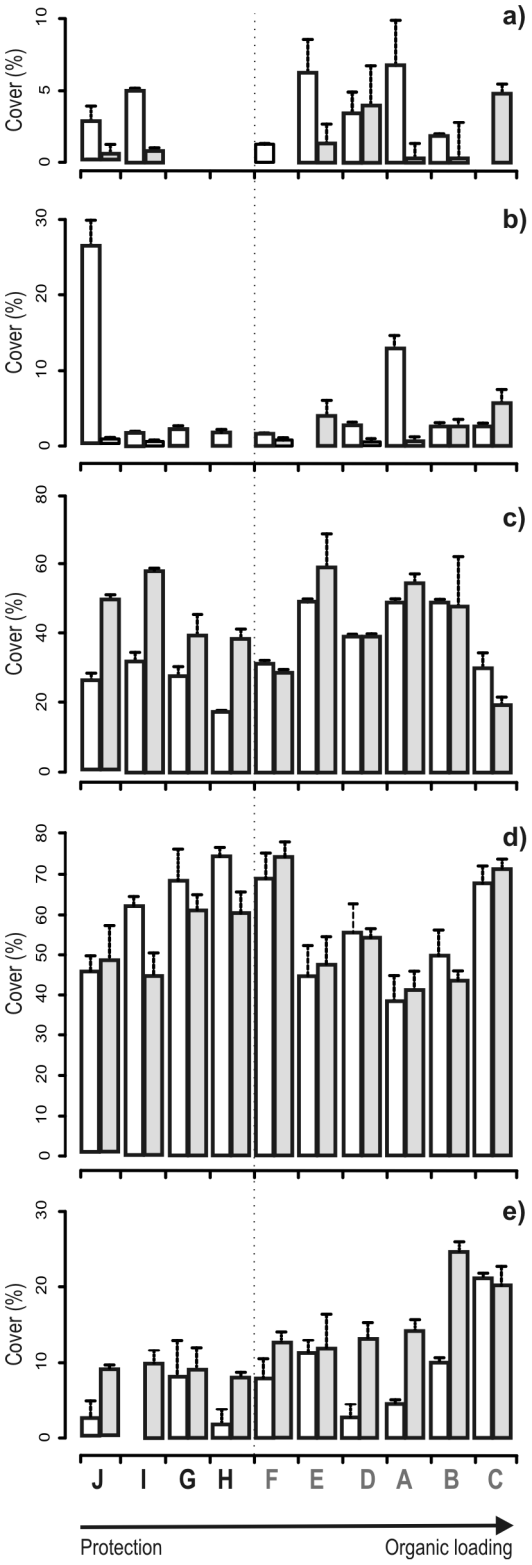
Cover of three dimensional categories for each sites in 1993 (white) and 2008 (grey). **a**): high; **b**) medium-high; **c**) medium; **d**) medium-low; **e**) low. Note that scales on Y axis are different. The vertical dotted line separates the sampling sites within the MPA (black characters) from those outside (grey characters).

Considering the change in the percentage cover of three-dimensionality categories, all sites within the MPA experienced a reduction of the cover by the categories of ‘high’ or ‘medium-high’ three-dimensionality and a parallel increase of the cover of the category ‘medium’ three-dimensionality (see Figure 3).

Sea urchins were represented in both years by *Paracentrotus lividus* and, especially, *Arbacia lixula*. There were no obvious changes in their densities between 1993 and 2008. Sea-urchins were absent from most quadrats in both years, with few exceptions. The highest sea urchin density in 1993 was recorded at site J (4 ind. m^−2^±2.5 SE) and in 2008 at site E (3 ind. m^−2^±1.1 SE).

## Discussion

In this study we found that an MPA and a network of six SCIs, two major tools used in the Mediterranean Sea for EBM implementation, were ineffective in halting degradation on rocky reef sessile communities. Species composition and architectural complexity changed significantly between 1993 and 2008, before and after the implementation of these conservation measures. Although we compared only two points in time, too few to assess long-term change and to elucidate cause-effect relationships, the strong association of the observed changes in the communities with the documented pressure regime allows generating hypotheses about possible drivers of change. In particular, our analyses indicate that organic loading from sewage outfalls may be a key driver of the observed change in these communities.

**Figure 4:**
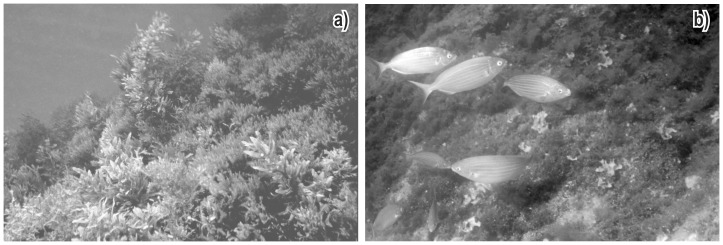
Change in reef community at Portofino, in the vicinity of sampling site J: a) *Sargassum vulgare* and *Dictyopteris polypodioides* canopy in 1981; b) *Sarpa salpa* grazing in a turf-dominated environment in 2009.

The taxonomic composition of rocky reef communities within the MPA became more similar, in 2008, to that of the communities close to sewage outfalls than in the past, thus implying a reduction of β diversity over the whole area. Three-dimensional structure decreased owing to the reduction of erect forms (e.g. tall macroalgae) and a parallel increase of flatter forms (e.g. turf-forming or encrusting organisms). In 1993, assemblages within the MPA and far from sources of impact were dominated by erect algae, most of which were canopy-formers. Earlier descriptive information from the 1950s through the 1980s [18], [33]–[38] reported the diffuse presence of dense canopies of the brown algae *Dictyopteris polypodioides* and *Sargassum vulgare* (Figure 4), already rare in 1993 and virtually absent in 2008. The loss of habitat complexity is a sign of degradation that has been highlighted as one of the major threats to marine biodiversity worldwide [8], [12]. Notwithstanding sessile species diversity showed slightly higher in 2008 than in 1993, reduced habitat complexity is likely to have negatively affected numerous canopy-associated epiphytic and motile organisms, whose diversity and abundance is known to be facilitated by habitat cascades via habitat provision and ecosystem engineering [39], [40]. Moreover, we cannot discard the possibility that the occurrence of algal canopies in 1993 hid species that showed obvious only to the visual census of 2008, when canopies were reduced.

In temperate reefs, sea water warming and associated extreme climate events are known to be able to cause extirpation of canopy-forming macroalgae [41]–[43]. However, lack of erect macroalgae from unprotected sites was already noticed in 1993, i.e. before the heat waves of 1999 and 2003. Similarly, the arrival in the Ligurian Sea of the invasive species *Acrothamnion preissii* and *Caulerpa racemosa* predates the heat waves [44], [45]; establishment of invasive species is said to be favored by already stressed ecosystems [46], [47]. A shift from erect-dominated to turf-dominated algal communities can be produced by nutrient enrichment from sewage outfalls [48]. Our sampling sites were relatively far from sewage outlet; therefore, no indicator species of organic pollution was detected. However, algal turf and many of the algal species first appeared or found more abundant in 2008 are known to be comparatively tolerant to lowered water quality with respect to those dominating in 1993 [49].

In the Tigullio Gulf, the undiminished input of organic load has possibly expanded its effects further away from its sources in recent years. The assemblages close to these chronic sources already showed a bi-dimensional structure in 1993 and have likely changed less as compared to the furthest sites, still architecturally complex in 1993. The sequence of disturbance is known to affect the final structure of communities whose response depends also on their previous history [50]. The spatio-temporal variation of stressors intensities is likely to be the main driver of the delay in degradation observed in the MPA. However, the establishment of the MPA in 1998 and the implementation of SCIs were ineffective in reversing the trend toward the deterioration of communities. Finding the alien alga *Caulerpa racemosa* in 2008 constituted a further signal of alteration. *C. racemosa* has been recognized as a strong invader [51] and its fast spread is favored by the presence of algal turf [52]. Turf development in the protected sites anticipated the settlement of *C*. *racemosa*, which has been subsequently recorded also in the Portofino MPA [53].

Our results suggest sequential community change under the combined influence of human pressure and invasive species. Reef communities are undergoing a shift from canopy-dominated to turf-dominated, and then to a *C. racemosa*-dominated state. This trajectory towards ecosystem deterioration has not been hampered by protection measures. Paradoxically, protection itself might even have contributed to the observed community shift. Fishing restriction through MPA enforcement may have a dual effect on reef communities: on one hand, it recovers the abundance of large carnivorous fish, which have the desirable effect of controlling sea-urchin overgrazing [54]; on the other hand, it also favors large herbivorous fishes such as *Sarpa salpa*. Sea urchin densities did not change between 1993 and 2008 and, although published quantitative data about their densities prior to 1993 are not available, field observations in the 1980s by two of us (CNB and CM) suggest no obvious change with time. On the contrary, the abundance of *S. salpa* was shown to have increased after the creation of the Portofino MPA [15]. Increased grazing by *Sarpa salpa* is capable of driving algal assemblages towards a turf-dominated state [55].

Anthropogenic stressors and climate change may act in synergy on temperate reef communities, lowering their resilience to perturbations [56]. While climate and other global impacts require international actions, regional management practices may help reducing local impacts. Managing temperate reefs requires efforts to reduce nutrient and organic inputs from land sources and pollution by boat traffic. Best coastal management practices may promote favorable conditions to confer canopy-forming species with resistance and resilience against pressures that cannot be managed locally, such as sea water warming, storms, and introduced species.

**Figure 5:**
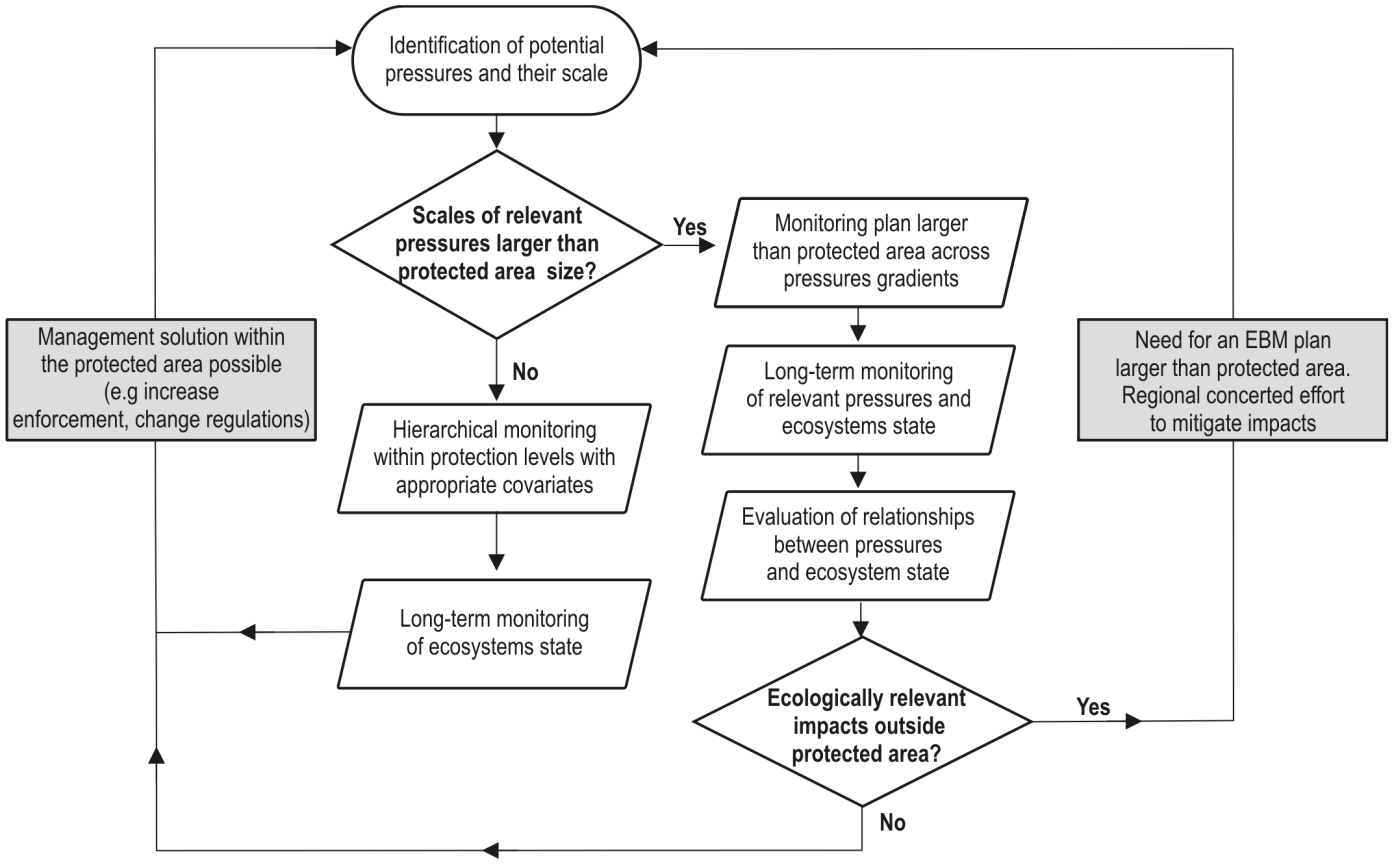
Decision flow-chart for implementing EBM and choosing effective monitoring design in coastal zones to be protected. White boxes: steps required for ecosystem based evaluation of relevant pressures; grey boxes: policy consequences of ecosystem-based analysis.

MPAs, which are designed mainly to regulate fishing activities, are largely ineffective in managing land-based pressures (e.g. outfalls, river discharge) and the spread of invasive species. In this context, SCIs or other complementary measures need to play a significant role in addressing diverse and escalating threats. Yet, SCIs implementation regulated a limited set of pressures (i.e. anchoring and the building of new coastal structures) that may not be the major drivers of change, especially in cases as the Tigullio Gulf where multiple and chronic pressures act together. At present, SCIs represent a ‘passive’ application of the legislative constraints in the coastal zones. Legislation, of course, plays a crucial role for conservation. However, legislative constraints alone are not enough to achieve effective EBM [3]: managers and scientists should apply and use the legislative instruments in a scientifically sound way. For SCIs to become effective EBM tools, the selection of the crucial pressures to be managed should not be driven by existing laws, but should rather be dictated by the actual pressure regime and local ecological characteristic of different coastal zones (Figure 5).

Small-scale protection measures and SCIs will not be effective if key large scale pressures, such as those coming from adjacent unprotected zones or linked to global change, are not identified. Comprehensive management plans should address multiple pressures through a suite of policy and management tools: carefully designed monitoring is critical for the early detection and eradication of invasive species, for the upgrade of water treatment plants, and for the regulation of coastal development in proximity to sensitive reef and seagrass habitats.

Monitoring programs that are too narrow in scope, e.g., involving only ecosystems within MPAs, may fail in the identification of relevant pressures that often act at larger scales [57] and in the quantification of the cumulative impacts of multiple pressures [1], [7]. Only when pressures are exclusively local, monitoring based on hierarchical designs within the protected area and according to enforcement levels may be effective in providing managers with valuable solutions [58]. When multiple pressures originate outside the boundaries of the protected area and have wider impact, monitoring programs should be designed across gradients at the appropriate scales to address relevant pressures and resulting cumulative impacts (Figure 5). This will help identifying the ultimate causes of deterioration and setting management priorities to reverse ecosystem trajectory.

## Supporting Information

Table S1 Characteristics of the main pressures in the study area. Information on beach replenishment was available from 2003.
(DOCX)

Table S2 Significant correlations of the individual pressures with the first three axes of the Principal Component Analysis (PCA).
(DOCX)

Table S3 Results of SIMilarity PERcentage (SIMPER) analysis identifying taxa major contributing to differences between Years and among Sites. Grey letters indicate sampling sites outside the MPA while black letters indicate sampling sites within the MPA boundaries.
(DOCX)

Figure S1 **a**) Annual and seasonal mean (+SE) of chlorophyll-a concentration in the period 1985–1994 (grey bars) and in the period 2002–2008 (black bars). **b**) Annual and seasonal mean (+SE) of water transparency (measured with Secchi disk) in the period 1985–1994 (grey bars) and in the period 2002–2008 (black bars). **c**) Potential organic load (expressed in term of equivalent inhabitants) calculated for the 7 administrations facing the coastal zone of the study area following the method described by Lopez y Royo et al. (2010); C: Camogli; L: Lavagna; P: Portofino; R: Rapallo; Sa: Santa Margherita; Se: Sestri Levante; Z: Zoagli; grey bars: data relative to 1991; black bars: data relative to 2001.
(TIF)
