# Supplementary material for: Conserving Biodiversity in a Human-Dominated World: Degradation of Marine Sessile Communities within a Protected Area with Conflicting Human Uses
Source: PLoS One. 2013 Oct 15;8(10):e75767. doi: 10.1371/journal.pone.0075767 (PMC3797118; doi:10.1371/journal.pone.0075767)
Supplement: Table S1 — Characteristics of the main pressures in the study area. Information on beach replenishment was available from 2003. (DOCX) [file pone.0075767.s001.docx]

**Table S1.** Characteristics of the main pressures in the study area. Information on beach replenishment was available from 2003.

| **beach replenishment** | **years** | **sediment used (m^3^)** | **coast length involved (m)** |
| --- | --- | --- | --- |
| **1** | 2003 | 7,200 | 400 |
| **2** | 2004 and 2005 | 45,222 | 3,000 |
| **3** | 2003 ÷ 2007 | 50,000 | 2,800 |
| **4** | 2003 and 2005 | 2,900 | 200 |
| **5** | 2004 ÷ 2006 | 1,211 | 578 |
| **6** | 2003 ÷ 2007 | 11,080 | 996 |
| **sewage outfalls** | **year of construction** | **capacity (m^3^·day^-1^)** | **inland treatment** |
| **1** | 1990 | 4,300 | Sedimentation |
| **2** | 1979 | unstated | Sedimentation |
| **3** | 1979 | 8 | Liquid O_2_ treatment |
| **4** | 1987 | 1,125 | None |
| **touristic marinas** | **year of construction** | **surface (m^2^)** | **number of boats** |
| **Portofino** | before 1970 | 30,000 | 253 |
| **Santa Margherita** | before 1970 | 100,000 | 700 |
| **Rapallo Riva** | 1970s | 54,000 | 376 |
| **Rapallo comunale** | before 1970 | 30,000 | 517 |
| **Chiavari** | 1970s | 53,000 | 459 |
| **Lavagna** | before 1970 | 290,000 | 1,455 |
| **Sestri Levante** | before 1970 | 72,000 | 454 |
