# Supplementary material for: Conserving Biodiversity in a Human-Dominated World: Degradation of Marine Sessile Communities within a Protected Area with Conflicting Human Uses
Source: PLoS One. 2013 Oct 15;8(10):e75767. doi: 10.1371/journal.pone.0075767 (PMC3797118; doi:10.1371/journal.pone.0075767)
Supplement: Table S2 — Significant correlations of the individual pressures with the first three axes of the Principal Component Analysis (PCA). (DOCX) [file pone.0075767.s002.docx]

**Table S2.** Significant correlations of the individual pressures with the first three axes of the Principal Component Analysis (PCA).

| **Pressure** | **PCA1** | **PCA2** | **PCA3** |
| --- | --- | --- | --- |
| Distance from MPA | 0.560 | 0.241 |  |
| Distance from outfall 1 | -0.609 |  |  |
| Distance from outfall 2 | -0.612 | -0.644 |  |
| Distance from outfall 3 | -0.629 | -0.154 |  |
| Distance from outfall 4 | -0.214 |  |  |
| Distance from river mouth | -0.632 | -0.163 |  |
| Distance from beach replenishment site |  |  | 0.997 |
| Distance from touristic marinas |  |  | 0.643 |
