# Supplementary material for: Conserving Biodiversity in a Human-Dominated World: Degradation of Marine Sessile Communities within a Protected Area with Conflicting Human Uses
Source: PLoS One. 2013 Oct 15;8(10):e75767. doi: 10.1371/journal.pone.0075767 (PMC3797118; doi:10.1371/journal.pone.0075767)
Supplement: Table S3 — Results of SIMilarity PERcentage (SIMPER) analysis identifying taxa major contributing to differences between Years and among Sites. Grey letters indicate sampling sites outside the MPA while black letters indicate sampling sites within the MPA boundaries. (DOCX) [file pone.0075767.s003.docx]

**Table S3.** Results of SIMilarity PERcentage (SIMPER) analysis identifying taxa major contributing to differences between Years and among Sites. Grey letters indicate sampling sites outside the MPA while black letters indicate sampling sites within the MPA boundaries

| **Taxa** | **sampling sites** | | | | | | | | | | | | | | | | | | | |
| --- | --- | --- | --- | --- | --- | --- | --- | --- | --- | --- | --- | --- | --- | --- | --- | --- | --- | --- | --- | --- |
|  | **A** | | **B** | | **C** | | **D** | | **E** | | **F** | | **G** | | **H** | | **I** | | **J** | |
|  | **93** | **08** | **93** | **08** | **93** | **08** | **93** | **08** | **93** | **08** | **93** | **08** | **93** | **08** | **93** | **08** | **93** | **08** | **93** | **08** |
| *Acetabularia acetabulum* | - | - | - | - | - | - | - | - | 8.9 | 4.9 | - | - | - | - | - | - | - | - | - | - |
| algal turf | - | - | - | 3.4 | 19.4 | 22.4 | - | - | - | - | - | 4.9 | - | 2.9 | - | - | - | - | - | - |
| *Caulerpa racemosa* | - | - | - | - | - | - | - | - | - | 4.3 | - | - | - | - | - | - | - | - | - | - |
| *Cladocora caespitosa* | - | - | - | - | 2.3 | 5.0 | - | - | - | - | - | - | - | - | - | - | - | - | - | - |
| *Cladophora prolifera* | 5.1 | 0.1 | 6.2 | 0.1 | 6.6 | - | - | - | - | - | - | - | - | - | - | - | - | - | - | - |
| *Codium bursa* | - | - | - | - | - | - | 8.9 | 2.7 | - | - | - | - | - | - | - | - | - | - | - | - |
| *Ellisolandia elongata* | 11.8 | 5.2 | - | - | - | - | - | - | - | 5.4 | - | - | - | - | - | - | - | - | 5.4 | 2.6 |
| *Crambe crambe* | 13.2 | 4.8 | - | - | 10.2 | 0.9 | - | - | - | - | - | - | 12.5 | 5.4 | - | - | 9.2 | 4.0 | 8.8 | 0.9 |
| *Cystoseira zosteroides* | 8.4 | - | - | - | - | - | - | - | - | - | - | - | - | - | - | - | - | - | - | - |
| *Dictyopteris polypodioides* | - | - | - | - | - | - | - | - | - | - | - | - | - | - | - | - | - | - | 25.0 | 0.2 |
| *Dictyota dichotoma* | 1.5 | 20.5 | 12.3 | 5.0 | - | - | 1.0 | 10.7 | 0.5 | 17.7 | 1.0 | 6.1 | 2.3 | 22.0 | 1.8 | 7.6 | 0.1 | 3.7 | 0.6 | 24 |
| *Dictyota implexa* | - | - | - | - | - | - | - | - | - | 12.0 | - | - | - | - | - | 4.3 | - | 6.9 | - | 10.6 |
| *Flabellia petiolata* | - | - | 5.6 | 7.8 | - | - | 8.7 | 11.6 | 8.6 | 2.9 | 13.4 | 6.6 | 14.1 | 1.9 | 4.9 | 3.5 | 8.9 | 9.9 | - | - |
| *Halimeda tuna* | - | - | - | - | 11.6 | 4.1 | - | - | 20.4 | 0.4 | 9.4 | 0.7 | - | - | - | - | 18.0 | 3.5 | - | - |
| Hydrozoa indet. | - | - | - | - | 0.7 | 5.8 | - | - | - | - | - | 2.9 | - | - | - | - | - | - | - | - |
| *Jania rubens* | - | - | 9.8 | 12.5 | - | - | 1.2 | 8.9 | 12.4 | 9.4 | 6.7 | 3.2 | 9.4 | 0.7 | - | - | - | 7.9 | - | - |
| *Laurencia obtusa* | - | - | - | - | - | - | - | - | - | - | - | - | - | - | - | - | - | - | 1.0 | 5.2 |
| *Lithophyllum incrustans* | - | - | 1.7 | 5.4 | 3.9 | 3.2 | - | - | - | - | - | - | - | - | - | - | 0.1 | 3.4 | - | - |
| *Mesophyllum lichenoides* | - | - | - | - | - | - | 11.3 | - | - | - | - | 22.7 | 3.5 | 14.0 | 24.3 | 24.7 | 4.4 | 2.6 |  |  |
| *Padina pavonica* | 18.1 | 11.5 | 18.3 | 6.7 | - | - | - | 6.1 | 14.6 | 7.0 | - | - | - | - | 2.9 | 5.5 | 0.9 | 8.6 | 3.9 | 4.2 |
| *Parazoanthus axinellae* | - | - | - | - | - | - | - | - | - | - | - | - | - | - | 4.3 | 0.8 | - | - | - | - |
| *Pennaria disticha* | - | - | - | - | - | 5.3 | - | - | - | - | - | - | - | - | - | - | - | - | - | - |
| *Peyssonnelia rubra* | - | 6.5 | - | - | - | - | - | - | - | - | 4.9 | 1.7 | - | - | - | - | - | - | - | - |
| *Peyssonnelia squamaria* | - | - | 12.3 | 5.0 | 14.6 | 23.9 | 24.4 | 7.3 | 8.6 | 5.6 | - | - | - | - | 7.8 | 2.9 | 7.2 | 8.0 | 5.8 | 11.5 |
| *Schizoporella errata* | - | - | - | - | 10.2 | 7.2 | - | - | - | - | - | - | - | - | - | - | - | - | - | - |
| *Sphaerococcus coronopifolius* | - | - | - | - | - | - | - | - | 7.0 | 1.5 | - | - | - | - | - | - | 4.7 | 0.9 |  |  |
| *Spongia lamella* | - | - | - | - | - | - | 4.2 | - | - | - | - | - | - | - | - | - | - | - | - | - |
| *Stypocaulon scoparium* | 6.8 | 6.2 | 18.2 | 20.4 | - | - | 4.4 | 4.8 | 3.2 | 14.3 | - | 5.2 | - | 6.3 | - | 10.9 | 1.2 | 18.0 |  |  |
| *Wrangelia penicillata* | - | - | - | - | - | - | - | - | - | - | - | - | - | - | - | - | - | - | 6.3 | 0.6 |
