# Supplementary figures and images for: Conserving Biodiversity in a Human-Dominated World: Degradation of Marine Sessile Communities within a Protected Area with Conflicting Human Uses
Source: PLoS One. 2013 Oct 15;8(10):e75767. doi: 10.1371/journal.pone.0075767 (PMC3797118; doi:10.1371/journal.pone.0075767)

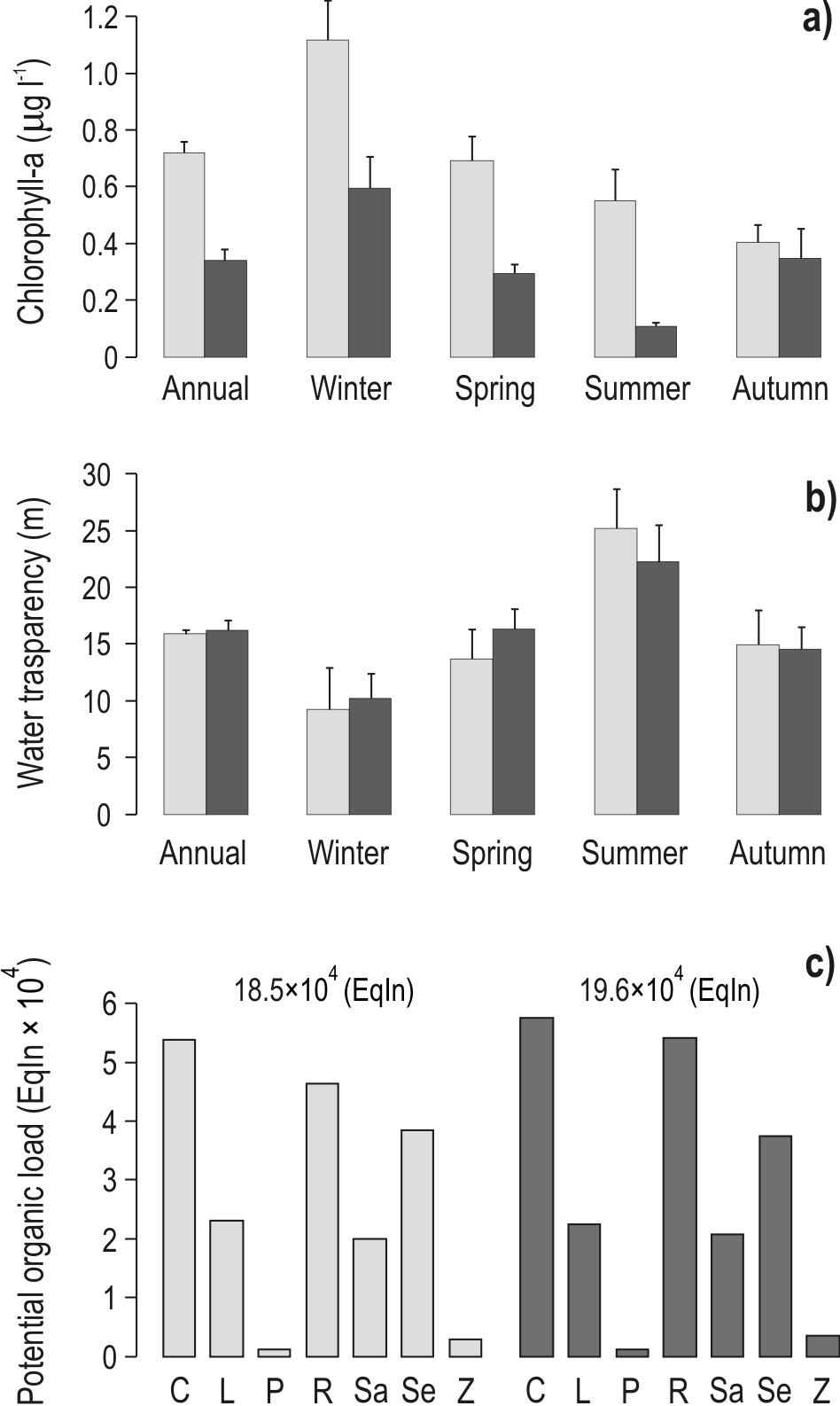

Supplement: Figure S1 — a) Annual and seasonal mean (+SE) of chlorophyll-a concentration in the period 1985–1994 (grey bars) and in the period 2002–2008 (black bars). b) Annual and seasonal mean (+SE) of water transparency (measured with Secchi disk) in the period 1985–1994 (grey bars) and in the period 2002–2008 (black bars). c) Potential organic load (expressed in term of equivalent inhabitants) calculated for the 7 administrations facing the coastal zone of the study area following the method described by Lopez y Royo et al. (2010); C: Camogli; L: Lavagna; P: Portofino; R: Rapallo; Sa: Santa Margherita; Se: Sestri Levante; Z: Zoagli; grey bars: data relative to 1991; black bars: data relative to 2001. (TIF) [file pone.0075767.s004.tif]
